# GlmS plays a key role in the virulence factor expression and biofilm formation ability of *Staphylococcus aureus* promoted by advanced glycation end products

**DOI:** 10.1080/21505594.2024.2352476

**Published:** 2024-05-13

**Authors:** Lijia Ni, Rui Shen, Hua Luo, Xuexue Li, Xiaofan Zhang, Lisi Huang, Yawen Deng, Xiaoyan Liao, Yonglin Wu, Chaohui Duan, Xiaoying Xie

**Affiliations:** a Department of Clinical Laboratory, Sun Yat-sen Memorial Hospital, Sun Yat-sen University, Guangzhou, China; b Institution of Antibiotic, Sun Yat-sen Memorial Hospital, Sun Yat-sen University, Guangzhou, China

**Keywords:** *Staphylococcus aureus*, advanced glycation end products, virulence factor expression, biofilm formation, GlmS-*sigB* regulatory axis

## Abstract

*Staphylococcus aureus (S. aureus)* is well known for its biofilm formation ability and is responsible for serious, chronic refractory infections worldwide. We previously demonstrated that advanced glycation end products (AGEs), a hallmark of chronic hyperglycaemia in diabetic tissues, enhanced biofilm formation by promoting eDNA release via *sigB* upregulation in *S. aureus*, contributing to the high morbidity and mortality of patients presenting a diabetic foot ulcer infection. However, the exact regulatory network has not been completely described. Here, we used pull-down assay and LC-MS/MS to identify the GlmS as a candidate regulator of *sigB* in *S. aureus* stimulated by AGEs. Dual-luciferase assays and electrophoretic mobility shift assays (EMSAs) revealed that GlmS directly upregulated the transcriptional activity of *sigB*. We constructed NCTC 8325 ∆*glmS* for further validation. qRT-PCR analysis revealed that AGEs promoted both *glmS* and *sigB* expression in the NCTC 8325 strain but had no effect on NCTC 8325 ∆*glmS*. NCTC 8325 ∆*glmS* showed a significant attenuation in biofilm formation and virulence factor expression, accompanied by a decrease in *sigB* expression, even under AGE stimulation. All of the changes, including pigment deficiency, decreased haemolysis ability, downregulation of *hla* and *hld* expression, and less and sparser biofilms, indicated that *sigB* and biofilm formation ability no longer responded to AGEs in NCTC 8325 ∆*glmS*. Our data extend the understanding of GlmS in the global regulatory network of *S. aureus* and demonstrate a new mechanism by which AGEs can upregulate GlmS, which directly regulates *sigB* and plays a significant role in mediating biofilm formation and virulence factor expression.

## Introduction

*Staphylococcus aureus (S. aureus)* is a commensal gram-positive pathogen that causes a range of infections in humans, from skin infections to life-threatening illnesses such as pneumonia, osteomyelitis, and sepsis [1]. *S. aureus* infections are more challenging to treat than non-biofilm-forming bacteria because of biofilm formation [2].

Diabetic foot ulcer infection (DFI) is a notorious consequence of *S. aureus* infection and biofilm formation [3]. As the predominant pathogen involved in the pathogenesis of DFI, *S. aureus* produces multiple virulence factors and forms biofilms, making the infection difficult to cure and leading to the high morbidity and mortality of patients presenting a diabetic foot ulcer infection [4]. The specific host factors of diabetic patients act as environmental stimuli, affecting *S. aureus* sessile biofilm growth. Advanced glycation end products (AGEs), a hallmark of chronic hyperglycaemia in diabetic tissues, have received increasing attention in recent years for their damaging role in diabetic pathogenesis [5–7]. AGEs are formed by the Maillard reaction, which occurs in an irreversible manner between amine group compounds (proteins, lipids, and nucleic acids) and carboxides (reducing sugar groups). Once formed, AGEs can continuously accumulate in biological tissues and produce incessant effects on the cells and microorganisms in the wound, even if blood sugar levels are well controlled. We previously demonstrated that AGEs significantly enhanced the biofilm formation of *S. aureus* by increasing extracellular DNA release. Global regulator sigma factor σB (SigB) plays an important role in the promotion of biofilm formation by AGEs through the downstream factor *lrg* operons [8]. Nevertheless, the regulatory network underlying biofilm formation by *S. aureus* is extremely complicated and poorly understood. Despite the unambiguous role of *sigB* activation by AGEs in biofilm formation, how AGEs act on *sigB* is not known.

In this work, we show that AGEs do not directly regulate *sigB* but instead activate the expression of *glmS*. To identify candidate regulators of *sigB* under AGE stimulation, we used pull-down assays and LC-MS/MS to show that the most significantly different protein pulled down by the SigB promoter probe was the glucosamine-6-phosphate synthetase (GlmS) protein. The dual-luciferase assay and electrophoretic mobility shift assay (EMSA) analysis confirmed that GlmS directly upregulates the transcriptional activity of *sigB*. We used quantitative RT-PCR (qRT-PCR) to show that AGEs promoted the expression of *glmS*, and the strains lacking *glmS* failed to highly express *sigB* or form biofilms in the presence of AGEs, indicating that GlmS and *sigB* constitute a regulatory pathway.

GlmS, discovered in 1991 by Baev et al. [9], is generally known as a ribozyme that can catalyse the conversion of fructose-6-phosphate and glutamine into glutamate and glucosamine-6-phosphate (GlcN6P), the starting point for bacterial cell wall synthesis. Aggregated GlcN6P can bind to the *glmS* ribozyme domain, activate latent self-cleavage activity, and degrade *glmS* mRNA, which is the foundation of *glmS* riboswitches [10]. This negative-feedback loop has become a new biomolecular target for the development of antibiotics and chemical/biological tools [11]. Previous studies regarding *glmS* of *S. aureus* have therefore focused on cell wall synthesis and drug resistance, along with the discovery of new riboswitch activators [12,13]. This study revealed another important role of GlmS involving global regulation through *sigB*, including biofilm formation and virulence factors. This is the first study to identify the crucial role of GlmS in the virulence factor expression of *S. aureus* and a new potential activator, AGEs. Consequently, another new regulatory pathway involving *sigB* activity has been found, which has improved the understanding of the global regulatory network of *S. aureus*.

## Materials and methods

### Bacterial strains, strain culture, and plasmid construction

*S. aureus* NCTC 8325, a type strain of *S. aureus*, was obtained from the China Medical Culture Collection Center (CMCC) and stored at −80°C. *Escherichia coli* DH5α was purchased from Solarbio, China (C1100) and used for plasmid construction.

NCTC 8325 colony was incubated overnight at 37°C with TSB broth in a shaker rotational speed of 200 r/min. All experiments were set up with experimental groups and blank groups. The experimental groups were stimulated with 50 µg/mL AGEs in TSB broth and cultured for 24 h. AGEs were purchased from Abcam, USA (ab51995). The blank groups were stimulated with the same concentration of bovine serum albumin (BSA) (EZ7890B203, BioFroxx, Germany).

The SigB promoter sequence was transferred into pGL4.10 vector (YouBio, China, VT1558) by restriction endonuclease sites XhoI and HindIII to express SigB promoter. The *glmS* gene CDS region was transferred into pcDNA3.1 and pET-28a vectors (YouBio, China, VT1001, VT1207) by restriction endonuclease sites XhoI and BamH I to express GlmS protein. The recombination products were transformed into competent *E. coli* DH5α cells and BL21(DE3) cells. Recombinant positive plasmids of pGL4.10 and pcDNA3.1 vectors were screened through ampicillin containing medium, while recombinant positive plasmids of pET-28a vector were screened through kanamycin containing medium.

### Pull-down and LC-MS/MS analysis

The pull-down assay utilized the natural affinity of streptomycin and biotin and was performed to analyse the interaction between DNA and proteins as described previously [14,15]. The SigB promoter was amplified by PCR using primers F (5’-CAGCTATGACCATGATTACGAATTCTTGCAAACGACAAAATTGATAAGTGCAATTAAATAAATGTTAGTAAG-3’) and R (5’-GTAAAACGACGGCCAGTGCCAAGCTTTATTTCGCACCTGCTCTTTTTTTATATACTTAGTCATACTGAT-3’). About 800 μl of nucleoprotein extracted from NCTC 8325 was premixed with 4 μg of biotin-labelled SigB promoter probe (Figure S2 and S3) and then 40 μl of streptavidin-agarose beads (Sigma, USA) were added and rotated at 4°C for 2 h. The beads were washed four times to collect pulled-down proteins and then boiled at 100°C for 10 min for further SDS-PAGE analysis. The protein bands in SDS-PAGE gel were visualized by silver staining. Shotgun proteomics, one of LC-MS/MS analysis (Thermo Scientific^TM^ Q Exactive^TM^, USA) was chosen to identify two groups of pulled-down proteins, and the protein peak maps were compared in the UniProt database (https://www.uniprot.org/taxonomy/1280). The mass spectrometry proteomics data have been deposited to the ProteomeXchange Consortium via the PRIDE [16] partner repository with the dataset identifier PXD046076. Gene Ontology (GO) functional analysis and Kyoto Encyclopedia of Genes and Genomes (KEGG) pathway analysis were performed on the differential proteins to help select candidate protein.

### Transfection and dual-luciferase reporter assay

The plasmid expressing the SigB promoter (Figure S4) was constructed using pGL4.10 vector, which can also express luciferase. The plasmid expressing the GlmS protein (Figure S5) was constructed using pcDNA3.1 vector. HEK-293 T cells (ATCC®CRL-1573^TM^) were prepared with trypsin in 24-well plates before transfection [17]. After 24 h of incubation at 37°C, the cell confluency rate was estimated to be approximately 70–80%. The SigB promoter plasmid, GlmS plasmid, and Hieff Trans™ liposomal transfection reagent (40802ES03, Yeasen Biotech, China) were mixed in serum-free DMEM (PM150210, Procell, China) according to the transfection reagent manufacturer’s protocol. After thorough mixing, the mixture was incubated for 10–25 min at room temperature to form the DNA-Hieff Trans^TM^ complex. Then, the DNA-Hieff Trans^TM^ complex was transfected into prepared HEK 293T cells after another 48 h of incubation [17]. Putative binding function and the regulation effect of GlmS protein were determined using the Dual-Glo® Luciferase Assay system (Promega, USA) according to the manufacturer’s protocol in 96-well plates. GloMax® (Promega, USA) was used to measure the activity of firefly luciferase and Renilla luciferase. The ratio of luminescence from firefly luciferase to Renilla luciferase was calculated [18,19].

### Electrophoretic mobility shift assay (EMSA)

EMSA was performed according to previous studies with some modifications [20]. BL21(DE3) cells were transfected with the GlmS plasmid. The expressed proteins were extracted and purified using a Ni-NTA 6FF Sefinose^TM^ Resin Kit (C600332, Sangon Biotech, China). The purified GlmS protein concentration was 0.75 mg/ml for subsequent experiments. The 400 bp positive SigB promoter fragment in the dual-luciferase assay was truncated into four segments of 100 bp. Each 100 bp fragment was biotinylated as an EMSA probe. EMSA was performed using a chemiluminescent EMSA kit (Axl-EMSA-100, Axl-Bio, China) according to the manufacturer’s instructions. A total of 0.1 pmol of biotinylated probe was added to each reaction. Additionally, 3.75 μg of purified protein was used per reaction when needed. The complexes obtained were separated by electrophoresis on a 5% polyacrylamide gel and then electrophoretically transferred onto nylon membranes. A 254 nm UV wavelength of 120 mJ/cm [2] was selected, and crosslinking was performed for 60 s using a UV-light crosslinker (SCIENTZ 03-II, SCIENTZ, China). The bands were visualized using chemiluminescence detection.

### Mutant construction

*S. aureus* NCTC 8325 is an ideal *S. aureus* strain for genetic manipulation [21]. To further illustrate the role of GlmS in biofilm formation, a *glmS*-knockout mutant (NCTC 8325 Δ*glmS*) was constructed with pKOR1 plasmid through homologous recombination using seamless cloning [22]. Two pairs of primers (*glmS*-pKOR1-F/*glmS*-pKOR1-R, pKOR1-*glmS*-F/pKOR1-*glmS*-R) were designed for PCR amplification. Then, *glmS*- pKOR1 products and pKOR1-*glmS* products were seamlessly connected and transformed into *E. coli* DH5α cells. Knockout plasmid was transferred to RN4220 using 2.5kV electricity. RN4220*glmS*-pKOR1 was transferred to NCTC 8325 through phage transduction method. Finally, the loss of *glmS* gene was confirmed by PCR using primers *glmS*-JD-F/*glmS*-JD-R and *glmS*-ter-F/*glmS*-ter-R. The PCR product electropherograms are shown in Figure S6. All the sequences of primers were listed in Table S7. During the culture process, 50 mM of N-acetyl-D-aminoglucose (GlcNAc) was added to the culture medium [23].

To comprehensively illustrate the role of GlmS in biofilm formation, a *glmS* overexpressing strain was constructed with pCM plasmid (BIO SCI, China). Primers F (5’-TTAGGAGGATGATTATTAATGTGTGGAATTGTTGGTTATA-3’) and R (5’-GTGGTGGTGGTGGTGGTGTTCCACAGTAACTGATTTAGC-3’) were designed to amplify target gene *glmS*. Then, amplify overexpression vector framework using primers F (5’-CACCACCACCACCACCACTGAATTCGTAATCATGTCATAG-3’) and R (5’-ACCAACAATTCCACACATTAATAATCATCCTCCTAAGGTA-3’). Two PCR products are seamlessly cloned and then transferred into *E. coli* dc10b competent cells. The recombinant positive plasmids were screened through chloramphenicol containing medium. Finally, the overexpressing plasmid is transferred back to *S. aureus* NCTC 8325 using 2.3kV electricity. SDS-PAGE was used to confirm the overexpression of the GlmS protein (Figure S7).

### RNA extraction and qRT-PCR

Total RNA was extracted from *S. aureus* under different culture conditions using TRIzol reagent (Invitrogen, USA) and ultrasonic waves. The quality and quantity of RNA were detected by a Nanodrop 2000 spectrophotometer (Thermo Fisher Scientific, USA) [24]. cDNA was first synthesized using a HyperScript^TM^ First-Strand cDNA Synthesis Kit (APExBIO, USA) and detected with HotStart^TM^ 2×SYBR Green qPCR Master Mix (APExBIO, USA). Each step referred to the kit manufacturer’s protocol. qRT-PCR was performed to detect the expression of global regulators of biofilm formation (*sigB*) and the expression of haemolysin genes (*hla*, *hlb*, *hld*, *hlg*) in *S. aureus* strains. The primers are listed in Supplementary Table S6. The relative expression levels of the tested genes were normalized to that of 16S rRNA [20].

### Hemolysis assay

The haemolytic ability assessment was based on the description of Jiang et al. [25] with minor modifications. In brief, NCTC 8325 and NCTC 8325 ∆*glmS* strains were incubated at 37°C for 24 h at a rotation speed of 200 rpm overnight. After centrifugation at 10,000 rpm for 8 min, the supernatant was collected. A 6% human erythrocyte solution was used to evaluate the haemolysis ability of the strains. Then, 100 μl of supernatant was added to 900 μl of erythrocyte solution, incubated at 37°C for 30 min, and centrifuged at 2500 rpm for 10 min. TSB was used as a negative control, and 0.1% Triton-X 100 was used as a positive control. The absorbance of the supernatant (OD_405_) was measured using a Multiskan^TM^ FC Microplate Photometer (Thermo Fisher Scientific, USA).

### Growth curves

The bacterial growth curve was determined as previously described, with minor modifications [26,27]. Briefly, NCTC 8325 and NCTC 8325 ∆*glmS* were inoculated into TSB broth. A blank group and an experimental group were set up for each strain as described before. Then, the culture medium was incubated at 37°C and 200 rpm. The growth curves were constructed based on the OD_620_ at 0, 2, 4, 6, 8, 10, 12, 14, and 24 h.

### Quantification of biofilms

Biofilm formation was performed according to previous studies [28,29]. Briefly, the overnight bacterial culture was prepared to a turbidity of 0.5 McFarland standards. A 20 µl bacterial suspension and 180 µl TSB medium were added to a 96-well flat-bottomed microtiter plate with lids in triplicate. After 24 h of incubation at 37°C, the wells were washed with PBS three times, stained with 0.5% (w/v) crystal violet (CV) for 10 min, and washed with water again. Then, the biofilm-associated CV dye was solubilized with 30% glacial acetic acid for 15 min at room temperature. The OD_595_ of each well was measured.

### Confocal laser scanning microscopy (CLSM)

For CLSM analysis, *S. aureus* strains were cultured in confocal dishes according to previous studies [28,30]. After 24 h of incubation at 37°C, the nonadherent culture of each well was washed with PBS and stained with AO and EB solution (R20292, Yuanye, China) in a dark location for 15 min at room temperature. The stained biofilms were imaged via confocal microscopy (Olympus FV3000, Japan). The living bacteria were stained green, and the dead bacteria were stained orange.

### Scanning electron microscopy (SEM) analysis

For SEM analysis, the *S. aureus* strain of interest was cultured in 6-well cell culture according to previous studies [30,31]. After 24 h of incubation, the biofilms were washed twice with PBS to remove nonadherent bacteria and fixed with 2.5% glutaraldehyde at 4°C for 4 h. Then, the biofilm samples were dehydrated and dried in a critical point dryer. After coating with gold powder, the biofilms were observed by scanning electron microscopy (Hitachi, Japan).

### Statistical analysis

All data are representative of three independent experiments and expressed as the mean ± standard deviation (SD). Statistical significance was assessed using unpaired and paired two-tailed Student’s *t* test with GraphPad Prism 9.0 (USA). *p* < 0.05 was considered statistically significant. * *p* < 0.05, ** *p* < 0.01, *** *p* < 0.001, **** *p* < 0.0001, and ns represents no significance. Graphs were drawn with GraphPad Prism 9.0 and Adobe Illustrator (USA).

## Results

### GlmS is a candidate protein that can directly combined with SigB promoter stimulated by AGEs in S. aureus

**Figure 1.**
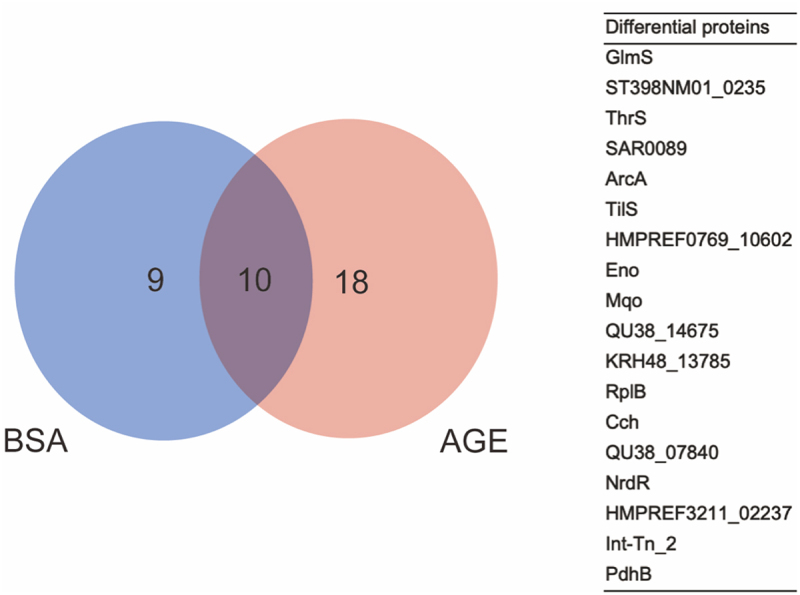
The differential proteins between BSA and AGEs groups. Left: venn diagram illustrating the candidate interacting proteins of the SigB promoter obtained after BSA and AGE treatment; right: a table of 18 differential proteins between BSA and AGEs groups.

The whole proteome and DNA pulled-down proteins of the NCTC 8325 strain were first separated by SDS-PAGE. The bands were visualized by silver staining to prove that we have pulled-down proteins for further identification and screening (Figure S1A). Then, shotgun proteomics LC-MS/MS analysis identified all of the pulled-down proteins, and we found 18 differential proteins (Figure 1). GO functional analysis revealed that GlmS, a metabolite interconversion enzyme, has catalytic activity and participates in cellular processes and metabolic processes [10,32–34]. KEGG pathway analysis indicated that GlmS may be related to the N-acetylglucosamine metabolism pathway, which can affect the cell wall synthesis of *S. aureus* [10,34,35]. Glms can recognize the small molecule in the environment, such as GlcN6P, and be activated to carry its physiologic functions [36]. Finally, GlmS protein was selected as a candidate protein.

### GlmS protein can directly bind to the SigB promoter and upregulate the transcriptional activity of sigB

**Figure 2.**
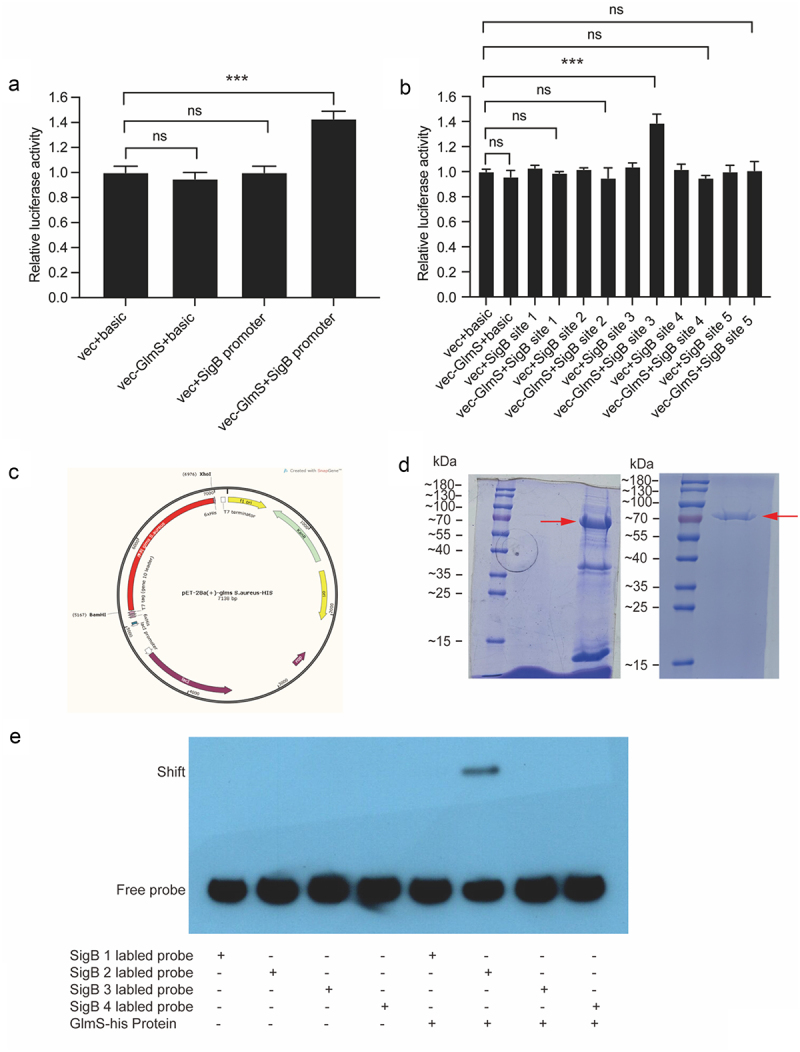
Verification of the binding sequence of the SigB promoter to the GlmS protein. (a) Luciferase activity of the SigB promoter in HEK-293T cells after cotransfection with GlmS-his. “vec” is short for “vector”. (b) Luciferase activity of five different fragments of the SigB promoter in HEK-293T cells after cotransfection with GlmS-his. (c) Vector map for expressing GlmS protein. (d) Left: SDS-PAGE electrophoresis of GlmS protein induced expression; right: SDS-PAGE electrophoresis of purified GlmS protein. The red arrow indicates the target protein, with a size of 72 KDa. (e) EMSA using different biotinylated probes of the SigB promoter to measure the binding ability of biotinylated probes with the GlmS protein. Lane 1, biotinylated probe 1 only; lane 2, biotinylated probe 2 only; lane 3, biotinylated probe 3 only; lane 4, biotinylated probe 4 only; lane 5, biotinylated probe 1 and GlmS protein; lane 6, biotinylated probe 2 and GlmS protein; lane 7, biotinylated probe 3 and GlmS protein; lane 8, biotinylated probe 4 and GlmS protein.

The dual-luciferase assay can indirectly evaluate the transcriptional activity of the target gene by quantitating both firefly and *Renilla* luciferases, commonly used in research on promoter transcriptional activity regulation [18,20] and miRNA target gene validation [37]. The result of dual-luciferase assay verifies that the GlmS protein can bind to the SigB promoter and upregulate its transcriptional activity (Figure 2a). To narrow down the binding sites between the two, the SigB promoter was divided evenly into five fragments. The dual-luciferase assay once again verified that GlmS protein can bind with the site 3 of SigB promoter and upregulate its transcriptional activity (Figure 2b). To further narrow down the binding sites between the two, site 3 was divided evenly into four fragments. GlmS protein was produced successfully and purified (Figure 2c and 2d), then used for EMSA. EMSA verified that probe 2 can bind to the GlmS protein (Figure 2e). Thus far, dual-luciferase reporter assay and EMSA determined the molecular mechanism by which GlmS protein can directly bind with the 100 bp fragment (5’-TTTTAACGGATGGTGTGACTGAAGCTAGAAATAGTGAAGGTACCTTTATAGATAAACAAAAACTTTTAGAATATATTAAAAAACATAAACATATGCACCC-3’) of SigB promoter and upregulate its transcriptional activity.

### AGEs upregulate the mRNA expression of glmS

The relative quantification of mRNA expression of *glmS* under the promotion of BSA and AGEs was evaluated by the 2^−∆∆CT^ method (Table 1). The average 2^−∆∆CT^ value indicated that the mRNA expression of *glmS* gene increased by 3.28 times under the promotion of AGEs compared to BSA. Moreover, a statistical analysis of paired *t*-tests was performed on ∆CTs of the BSA and AGEs groups, and there was a significant difference between the two (*p* < 0.05).

**Table 1.** Relative quantification of the mRNA expression of *glmS* under the promotion of BSA and AGEs using the 2^−∆∆Ct^ method.

	Average CT of BSA			Average CT of AGEs		
	1	2	3	1	2	3
16S rRNA	9.43	8.53	8.50	9.82	8.74	8.95
*glmS*	14.47	16.91	14.60	13.93	15.12	13.07
∆CT	5.04	8.38	6.10	4.11	6.39	4.12
∆∆CT*				−0.94	−1.99	−1.98
2^−∆∆CT^				1.91	3.98	3.94
Average 2^−∆∆CT^				3.28		

*∆∆CT = (Average CT of AGEs) – (Average CT of BSA).

### The glmS gene contributes to the nonpigmented phenotype, hemolytic phenotype, and cell wall synthesis of S. aureus

**Figure 3.**
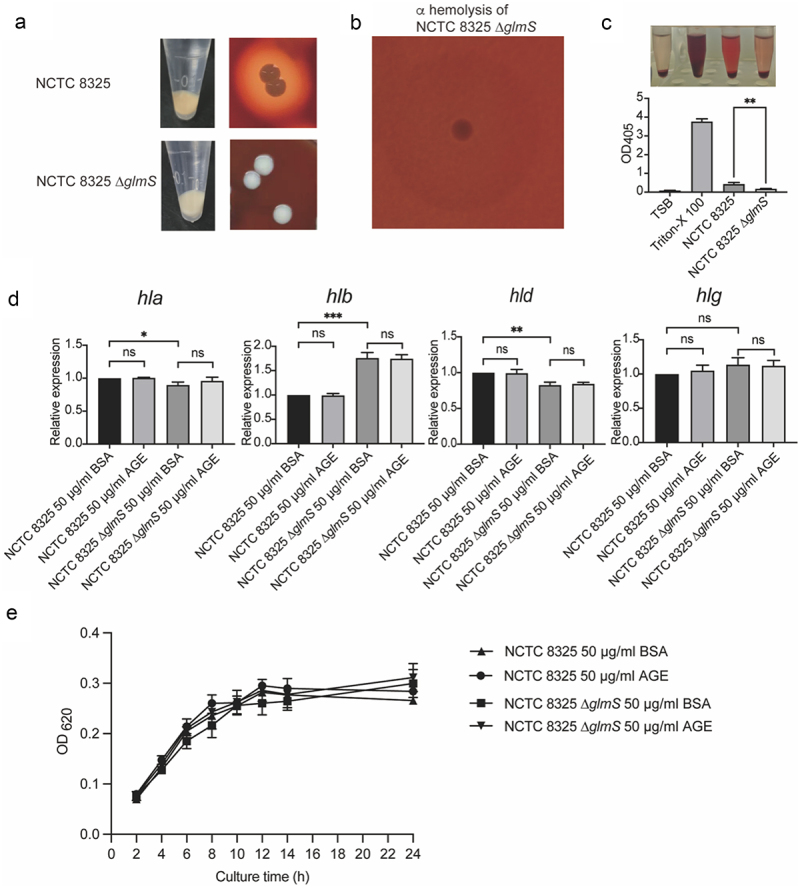
The growth and hemolytic activity of NCTC 8325 and NCTC 8325 ∆*glmS*. (a) Colony morphology and hemolysis of NCTC 8325 and NCTC 8325 ∆*glmS* cultured on Columbia blood agar. (b) The hemolytic ability changed from β hemolysis to α hemolysis. (c) The hemolytic activity of NCTC 8325 and NCTC 8325 ∆*glmS*. TSB was used as the negative control. Triton-X 100 (0.1%) was used as a positive control. (d) Expression levels of hemolysin genes (*hla*, *hlb*, *hld*, *hlg*) in NCTC 8325 and NCTC 8325 ∆*glmS* determined by qRT-PCR. Relative gene expression was indicated by 16S rRNA gene normalization. * *p* < 0.05, ** *p* < 0.01, *** *p* < 0.001, and ns represents no significance. (E) The growth curve of NCTC 8325 and NCTC 8325 ∆*glmS* promoted by BSA and AGEs.

**Figure 4.**
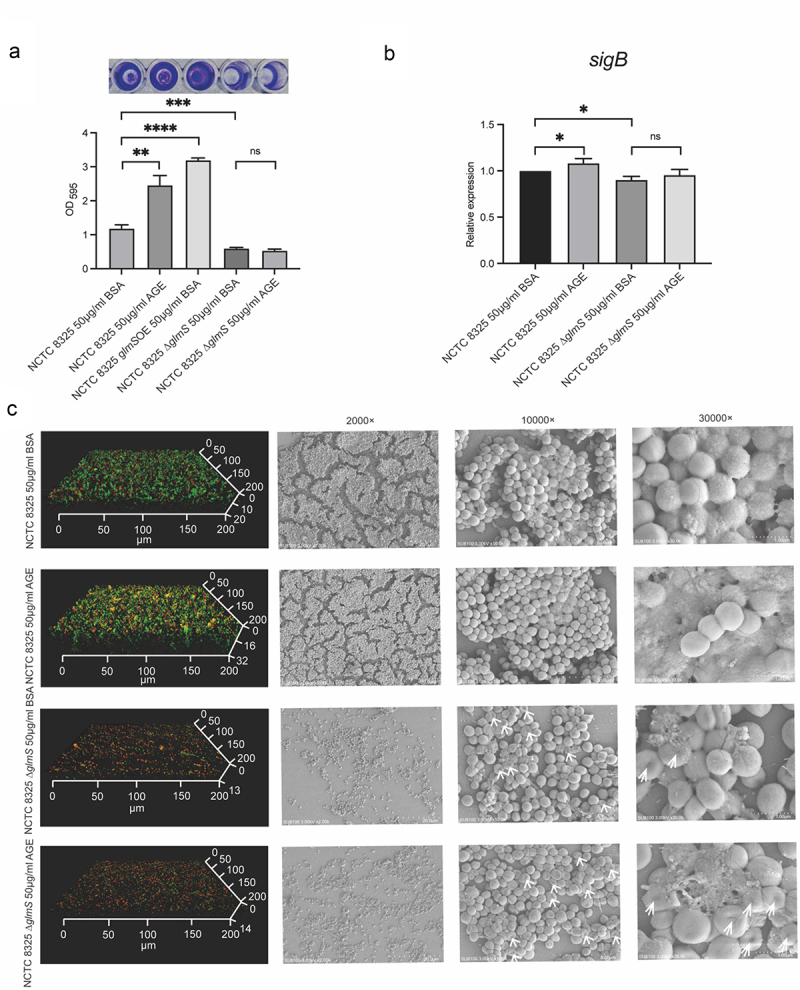
Biofilm formation and expression levels of *sigB* of NCTC 8325 and NCTC 8325 ∆*glmS* promoted by BSA and AGEs. (a) Crystal violet staining of the biofilms. (b) Expression levels of *sigB* in the NCTC 8325 and NCTC 8325 ∆*glmS* strains promoted by BSA and AGEs. (c) Images of biofilms obtained using CLSM and SEM. The living bacteria were stained green, and the dead bacteria were stained orange. * *p* < 0.05, ** *p* < 0.01, *** *p* < 0.001, **** *p* < 0.0001, and ns represents no significance. The white arrow indicates the cell wall depression of NCTC 8325 ∆*glmS*.

The NCTC 8325 strain has the typical colony morphology of *S. aureus*. The colony appears golden in colour, with a wide β haemolytic ring around the colony. However, NCTC 8325 ∆*glmS* lost its golden pigment, and the colony turned white (Figure 3a). Moreover, the haemolysis ability of NCTC 8325 also changed from β haemolysis to α haemolysis (Figure 3b), accompanied by a notable margin in haemolytic capacity (Figure 3c). In addition, the cell wall of NCTC 8325 ∆*glmS* showed a significant depression, as shown by the arrow (Figure 4c), which indicated once again that GlmS is the key ribozyme related to cell wall synthesis [10].

The growth curves of NCTC 8325 and NCTC 8325 ∆*glmS* demonstrated that BSA and AGEs do not impact their growth (Figure 3e), excluding the influence of growth factors on haemolysin gene expression and biofilm formation experiments.

### The glmS gene contributes to changes in hemolysin gene expression in S. aureus

After observing changes in the haemolytic phenotype of the knockout strain (Figure 3B), we further detected the expression of haemolysin genes using qPCR. Compared with NCTC 8325, NCTC 8325 ∆*glmS* showed downregulation of *hla* and *hld* expression and upregulation of *hlb* expression. Meanwhile, AGEs had no promoting effect on the expression of haemolysin genes (Figure 3d). The changes in haemolysin gene expression caused decreased haemolysis ability of the NCTC 8325 strain (Figure 3c), which is a virulence factor of *S. aureus*.

### AGEs cannot upregulate the expression of sigB and induce biofilm formation by NCTC 8325 ∆glmS

The biofilm mass was evaluated using crystal violet staining (Figure 4a). The biofilm mass of NCTC 8325 ∆*glmS* were significantly less than the wild strain, both in AGEs and BSA groups (*p* < 0.001). For NCTC 8325 strain, the biofilms formation in AGEs group was significantly higher than in the BSA group (*p* < 0.01). However, in NCTC 8325 ∆*glmS* strain, there was no significant difference between AGEs and BSA groups. The *glmS* overexpressing strain can promote the biofilm formation (*p* < 0.0001), similarly to the wild strain exposed to AGEs. Confocal laser scanning microscopy (CLSM) and scanning electron microscopy (SEM) showed the same results (Figure 4c). Confocal images showed that the biofilm of NCTC 8325 ∆*glmS* were obviously thinner and sparser than that of wild strain both in BSA (13 ± 1 µm vs. 20 ± 1 µm) and AGEs (14 ± 1 µm vs. 32 ± 1 µm) (*p* < 0.01) groups. The biofilm of NCTC 8325 strain promoted by AGEs (32 ± 1 µm) was significantly thicker and denser than the BSA group (20 ± 1 µm) (*p* < 0.05). While the biofilm thickness of NCTC 8325 ∆*glmS* has no significant difference between AGEs (14 ± 1 µm) and BSA (13 ± 1 µm) groups. SEM images show that the biofilm of NCTC 8325 ∆*glmS* was significantly reduced compared to the wild strain. AGEs can promote the biofilm formation of NCTC 8325 strain, but have no effect on NCTC 8325 ∆*glmS*. The expression of *sigB* was significantly reduced when the NCTC 8325 strain knocked out *glmS* gene (*p* < 0.05). The *sigB* expression of NCTC 8325 strain was upregulated in by AGEs (*p* < 0.05), while no change in NCTC 8325 ∆*glmS* strain (Figure 4b).

## Discussion

In recent years, AGEs have attracted the attention of researchers due to increasing evidence of their involvement in many diseases, such as diabetes, cardiovascular diseases, cancers, neurodegenerative diseases, kidney diseases, liver diseases, infertility, infectious diseases, and even infection with the SARS-CoV-2 virus [5–7,38–40]. Our previous studies showed that the presence of AGEs, a reflection of hyperglycaemia in diabetic tissues, significantly promotes *S. aureus* biofilm formation via eDNA release. These effects are the consequence of upregulating *sigB* transcription and the subsequent downregulation of its downstream gene, *lrgA* [8].

Alternative *sigB*, which primarily modulates the stress responses of several gram-positive bacteria, plays essential roles in regulating biofilm formation, virulence factor expression, and pigment synthesis in *S. aureus* [1]. The *sigB* operon of *S. aureus* represents a global regulatory system that enables the organism to deal with environmental stresses [29,41]. AGEs, an important factor in the environment of diabetic foot tissue, upregulated *sigB* transcription, independent of the two-component systems Agr and SarA examined in our previous work. In this study, we further clarified that *sigB* is modulated by GlmS and enhances biofilm formation in *S. aureus* under AGE stimulation. To identify the regulator of *sigB* in the process of biofilm formation stimulated by AGEs, we used pull-down assays and LC-MS/MS to show that the most significantly different protein pulled down by the SigB promoter probe was the GlmS protein. The dual-luciferase assay and EMSA analysis confirmed that GlmS directly upregulates the transcriptional activity of *sigB*, and the binding site between the two was narrowed down to a 100 bp fragment (5’-TTTTAACGGATGGTGTGACTGAAGCTAGAAATAGTGAAGGTACCTTTATAGATAAACAAAAACTTTTAGAATATATTAAAAAACATAAACATATGCACCC-3’). To confirm the exact effect of biofilm formation, NCTC 8325 ∆*glmS* was used for biofilm formation experiments under AGE stimulation. We conducted a comprehensive and multilevel analysis on the biofilm formation of NCTC 8325 and NCTC 8325 ∆*glmS* under AGE stimulation, including the total amount of biofilm (Figure 4a), the thickness and vitality of the biofilms, and the density of the biofilms and the amount of extracellular matrix (Figure 4c). Compared with the wild-type strain, NCTC 8325 ∆*glmS* no longer responded to AGE stimulation in terms of biofilm formation. Concurrently, AGEs had no upregulation effect on *sigB* in NCTC 8325 ∆*glmS*, which indicated that GlmS plays a significant role in the *S. aureus* biofilm formation promoted by AGEs.

This discovery is very unexpected and interesting. To date, research on *glmS* has mainly focused on cell wall synthesis, drug resistance, and the development of antibiotic targets [11,32,34], but no research has shown its roles in the global regulation and pathogenicity of *S. aureus* thus far. In previous studies, riboswitches have been classified as a family of 5’-untranslated mRNA regions mostly found in bacteria. The *glmS* riboswitch is unique among the family of riboswitches, as it is a ribozyme that undergoes self-cleavage upon binding to GlcN6P and is distributed in many important human pathogens, such as *Bacillus anthracis*, *S. aureus*, *and Clostridium botulinum* [42]. Due to the close relationship with GlcN6P and cell wall synthesis, *glmS* is a very attractive target for developing broad-spectrum antibacterial compounds [13,34]. In our study, the growth curve of NCTC 8325 ∆*glmS*, cultured with 50 mM of GlcNAc remained unchanged compared to that of the wild-type strain (Figure 3e). However, it was also found that the cell wall of NCTC 8325 ∆*glmS* exhibited a depression (Figure 4c). The change in the cell wall is likely to cause changes in the drug sensitivity of a series of antibiotics that target the cell wall, such as penicillin, cephalosporin, vancomycin, bacitracin, and fosfomycin. In addition, we discovered the important role of GlmS in *S. aureus* pathogenicity. *S. aureus* produces many virulence factors involved in pathogenicity, which could be important in deciding infectious outcomes. As an important virulence factor, staphyloxanthin can impair neutrophil killing and protect bacteria against the host innate immune system [43]. Our study found that *glmS* knockout resulted in decreased pigment expression in the NCTC 8325 strain. Along with the inhibition of the production of staphyloxanthin in NCTC 8325 ∆*glmS*, the pathogenicity of the other strains was also significantly decreased, including haemolysin phenotype changes, changes in haemolysin gene expression and reductions in biofilms. The above virulence phenotype attenuation in NCTC 8325 ∆*glmS* was accompanied by downregulation of the *sigB* gene compared with that in the wild-type strain, which indicated that GlmS participates in the global regulation of *S. aureus* by regulating the activities of *sigB*, including virulence factor expression, pigments, biofilms, etc.

*GlmS* responds to the intracellular concentration of a variety of metabolites and second messengers. The natural ligand of *glmS* is GlcN6P [36], a key precursor for the synthesis of amino sugars in bacterial and eukaryotic cell walls, which participates in the glycosyl transfer reaction in the synthesis of glycoproteins by adding glycosyl to the proteins and thus forming glycoproteins. Recent research shows that carba-sugars and carba-sugar analogs can activate the *glmS* riboswitch [13,35]. AGEs refer to a group of stable terminal products produced by the free amino groups of proteins, amino acids, lipids or nucleic acids, and the aldehyde groups of reducing sugars. As the end products of GlcN6P, AGEs-upregulated *glmS* seems to be a new “negative feedback” regulation of ribose switching within bacterial cells, which requires further research and confirmation.

In conclusion, this study clarified the mechanism and regulatory pathway by which AGEs promote the biofilm formation of *S. aureus*, and elucidates a new regulatory factor of *sigB* and reveals the important role of the GlmS protein in the global regulatory network of *S. aureus.*

## Supplementary Material

Supplemental Material

## Data Availability

The data generated during the study are available at the repository figshare at https://doi.org/10.6084/m9.figshare.25627158.v1. The raw data of mass spectrometry proteomics data have been deposited to the ProteomeXchange repository at http://www.ebi.ac.uk/pride, with the dataset identifier PXD046076.
