# Supplementary material for: GlmS plays a key role in the virulence factor expression and biofilm formation ability of Staphylococcus aureus promoted by advanced glycation end products
Source: Virulence. 2024 May 13;15(1):2352476. doi: 10.1080/21505594.2024.2352476 (PMC11095574; doi:10.1080/21505594.2024.2352476)
Supplement: Supplemental Material [file KVIR_A_2352476_SM1071.docx]

Supplementary Material

**1 Supplementary Data**

1.1 SigB probe sequence (SAOUHSC_02298 promoter)

TTGCAAACGACAAAATTGATAAGTGCAATTAAATAAATGTTAGTAAGTGAATCATAATTATCCTTGCTTAAGCATTTGCTTTGTAAGGGAAGTGAGGAGGCAACTAATCGTGGAAGAATTTAAGCAACATTATAAGGGTTTAATTGATGAAAGTTTAACGTGCCAAGATAAAGTAGAATTGATAAAAAAGTGTGAGAAATACACTGACGAAGTGATTCGTAAGGACGTCTTGCCTGAAGACATTGTCGATATTCACAAAAACTATATATTGACGTTAAACTTAACGCGTGAAGATGTGTTCAAGACATTAGATGTCTTACAAGAAATTTGGTTATAGTTATCGAGATTATCAAAGATTGGTAGATAAACTTCAAGTTCACGATAAAGAGATAGACTTAGCTTCTAGCTTACAACAAACAATGCTTAAAACAGATATTCCACAATTTGATAGTATTCAAATTGGCGTTATTTCAGTGGCGGCACAAAAAGTAAGTGGAGATTATTTTAATTTAATTGACCATAACGATGGCACAATGAGCTTTGCTGTTGCAGATGTCATTGGAAAAGGTATACCAGCTGCTTTAGCAATGAGTATGATAAAGTTTGGCATGGATTCTTATGGACACTCACAATTACCGAGTGATGGTTTAAAACGTTTAAATCGTGTTGTTGAAAAGAATATTAATCAAAATATGTTCGTCACAATGTTTTATGGTTTATATGAAGAAATGAACCATTTATTGTATTGTAGTTCAGCTGGTCATGAGCCTGGATATATTTATCGCGCTGAAAAAGAAGAATTTGAAGAAATTTCAGTTAGAGGTAGAGTGTTAGGAATCAGTTCACAAACACGATATCAACAACAAGAAATTCCAATATACCTTGATGATTTAATTATCATTTTAACGGATGGTGTGACTGAAGCTAGAAATAGTGAAGGTACCTTTATAGATAAACAAAAACTTTTAGAATATATTAAAAAACATAAACATATGCACCCACAAGATATTGTTCAAATTATCTATGAAGCAATTTTAAAGCTTCAAAACCCAAATAAAAAAGATGATATGACTATTTTGATTATAAAAAGAGTAAATTAATTTAAAAAAGAAGATTAGAAATTATTTCGATGGGTATATAATAATTTGAAATATAAATATGGTGGATACAGCGCTTAAAATGAAGATAAATATTTTTAATAAGTAGGAGTGTAATGAAATGAATCTTAATATAGAAACAACCACTCAAGATAAATTTTACGAAGTTAAAGTCGGTGGAGAATTAGATGTTTATACTGTGCCTGAATTAGAAGAGGTTTTAACACCTATGAGACAAGATGGAACTCGTGATATTTATGTTAATTTAGAAAATGTGAGTTATATGGATTCGACAGGTTTAGGTTTATTCGTAGGTACATTAAAAGCATTAAACCAAAATGATAAAGAACTATACATTTTAGGTGTGTCAGATCGTATCGGTAGACTATTTGAAATTACTGGTCTTAAGGATTTAATGCATGTTAATGAAGGAACGGAGGTCGAATAACATGCAATCTAAAGAAGATTTTATCGAAATGCGCGTGCCAGCATCGGCAGAGTATGTAAGTTTAATTCGTTTAACACTTTCTGGCGTTTTTTCGAGAGCTGGTGCTACATATGATGATATTGAAGATGCCAAGATTGCAGTTAGTGAAGCTGTGACAAATGCAGTTAAACATGCATACAAAGAAAATAACAATGTGGGCATTATTAACATATATTTTGAAATTTTAGAAGATAAAATTAAAATTGTTATTTCTGATAAAGGTGACAGTTTTGATTATGAAACAACTAAATCAAAAATAGGTCCTTACGATAAAGACGAAAATATAGACTTTTTACGCGAAGGTGGCCTAGGTTTATTTTTAATCGAATCTTTAATGGATGAAGTCACAGTATATAAAGAATCTGGTGTGACAATCAGTATGACTAAGTATATAAAAAAAGAGCAGGTGCGAAATA

**2 Supplementary Figures and Tables**

**2.1 Supplementary Figures**


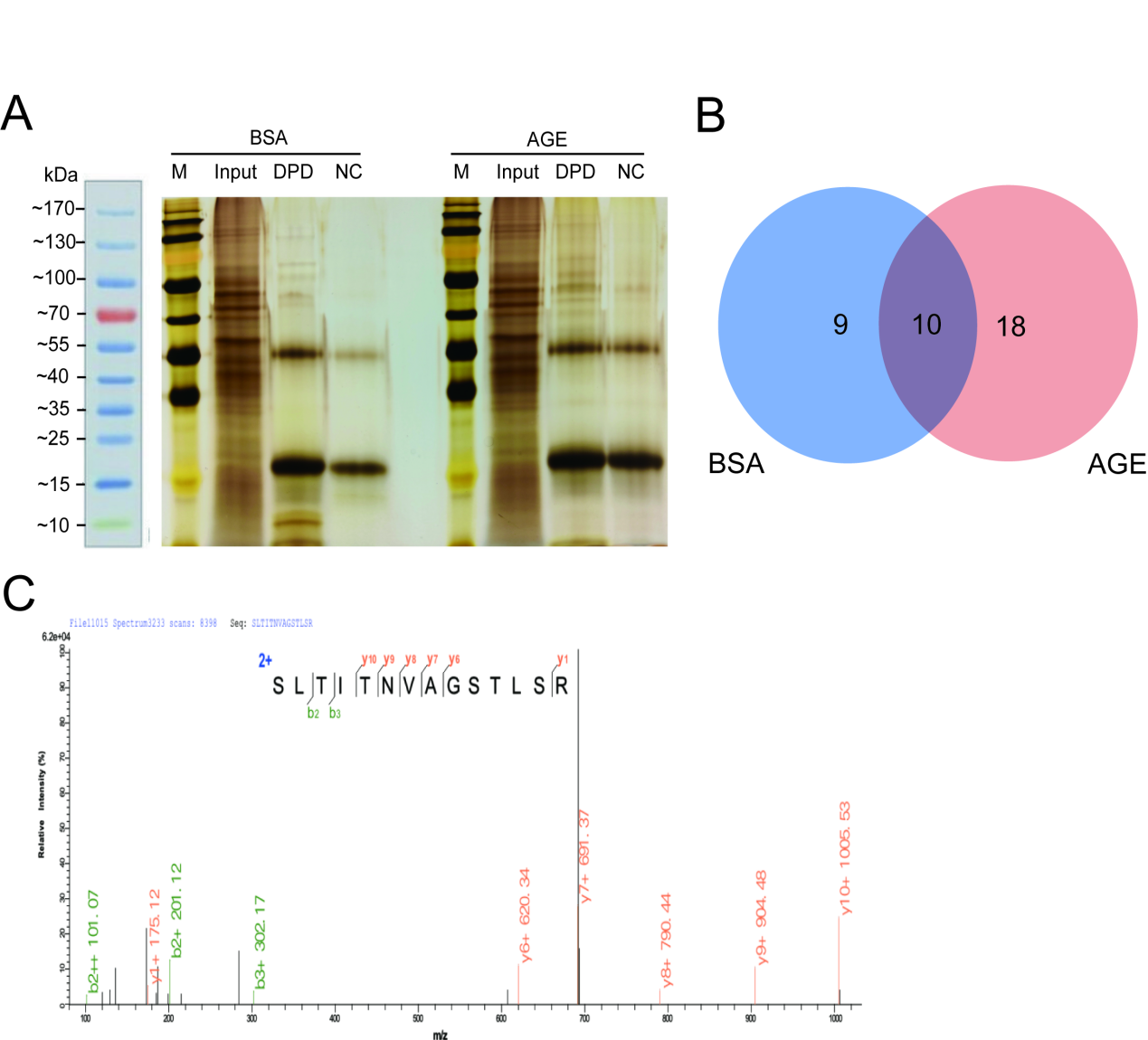


Supplementary Figure 1. Electrophoretic silver staining and differential protein identification of the pulldown experiment. (A) Silver staining of protein products after SDS‒PAGE. “M” represents “marker”. “Input” represents the whole proteome of NCTC 8325. “DPD” represents SigB promoter probe pulldown proteins. “NC” represents negative probe pulldown proteins. (B) Venn diagram illustrating the candidate interacting proteins of the SigB promoter obtained after BSA and AGE treatment. (C) Mass spectrometry identification peak of GlmS protein.


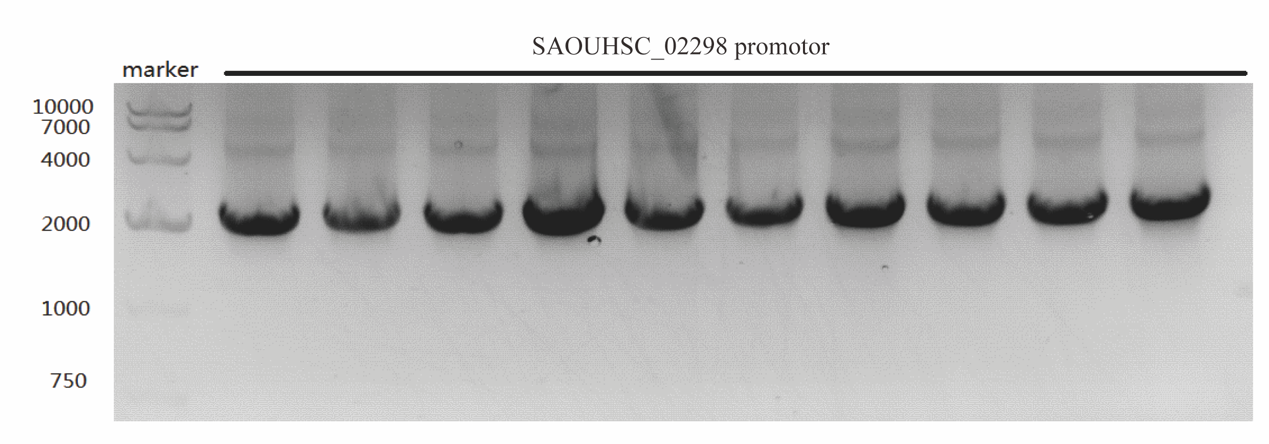


Supplementary Figure 2. Electropherogram of PCR products of SigB promotor.


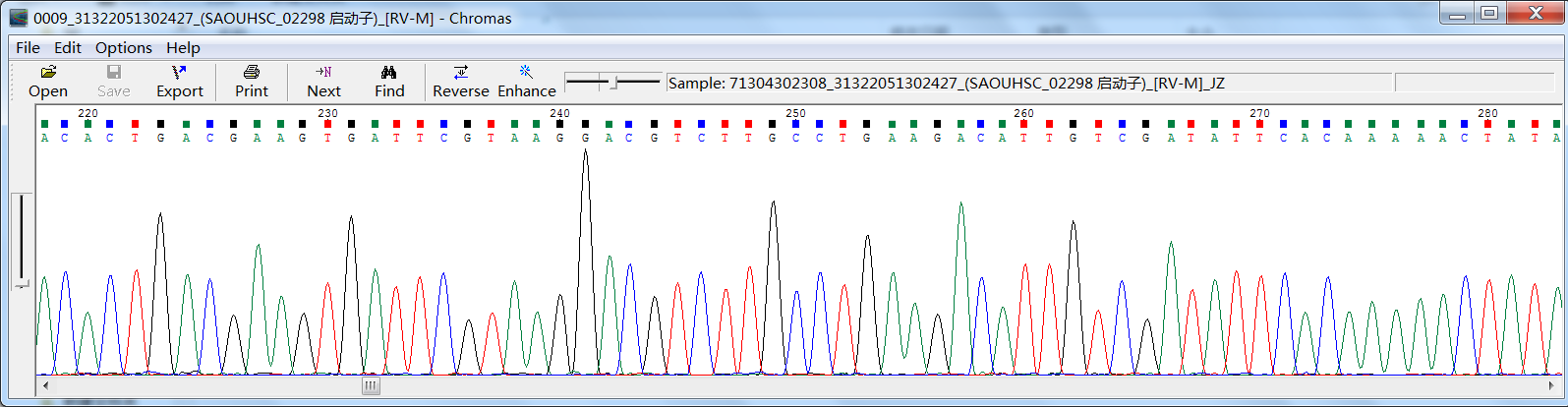


Supplementary Figure 3. Sequencing results of PCR products of SigB promotor.


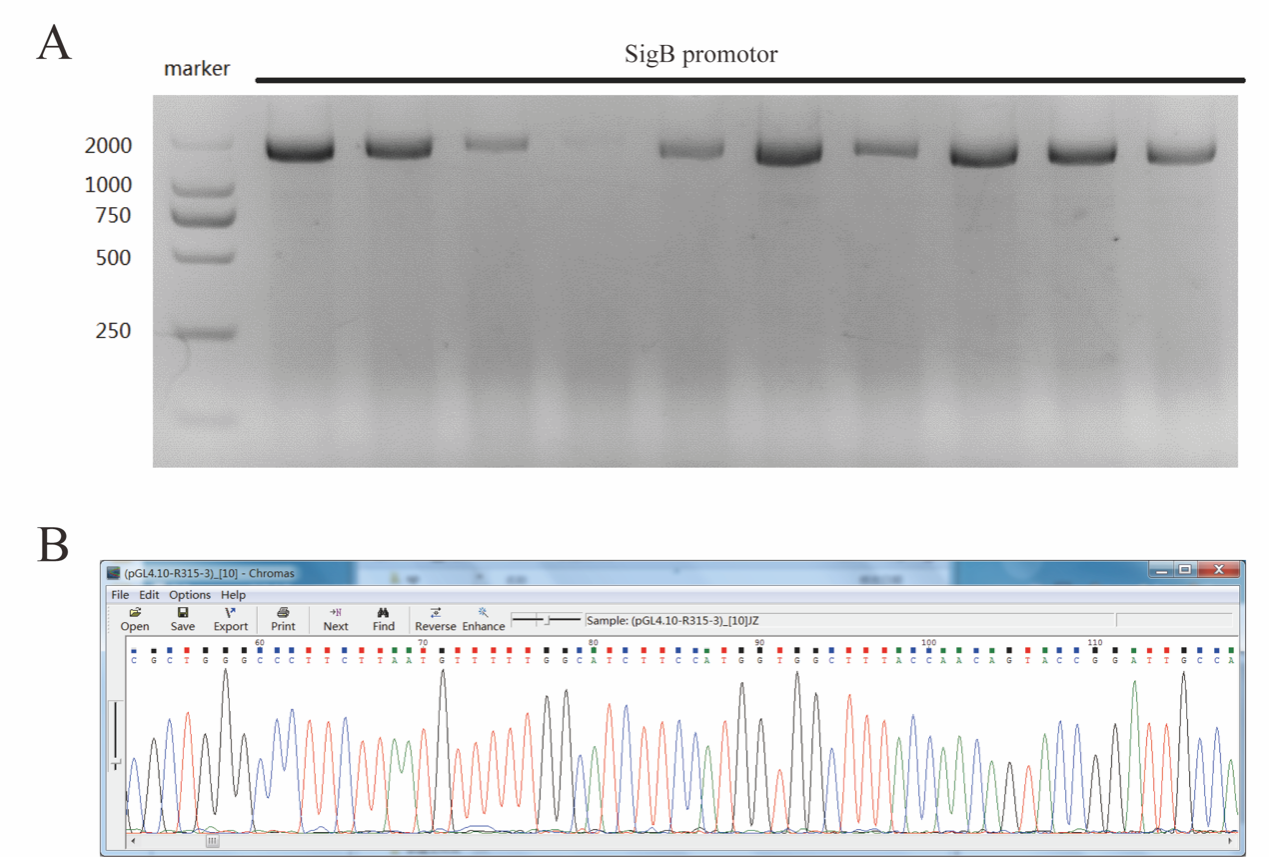


Supplementary Figure 4. Identifications of SigB promotor recombinant products. (A) Electropherogram of PCR products. (B) Sequencing results of PCR products.


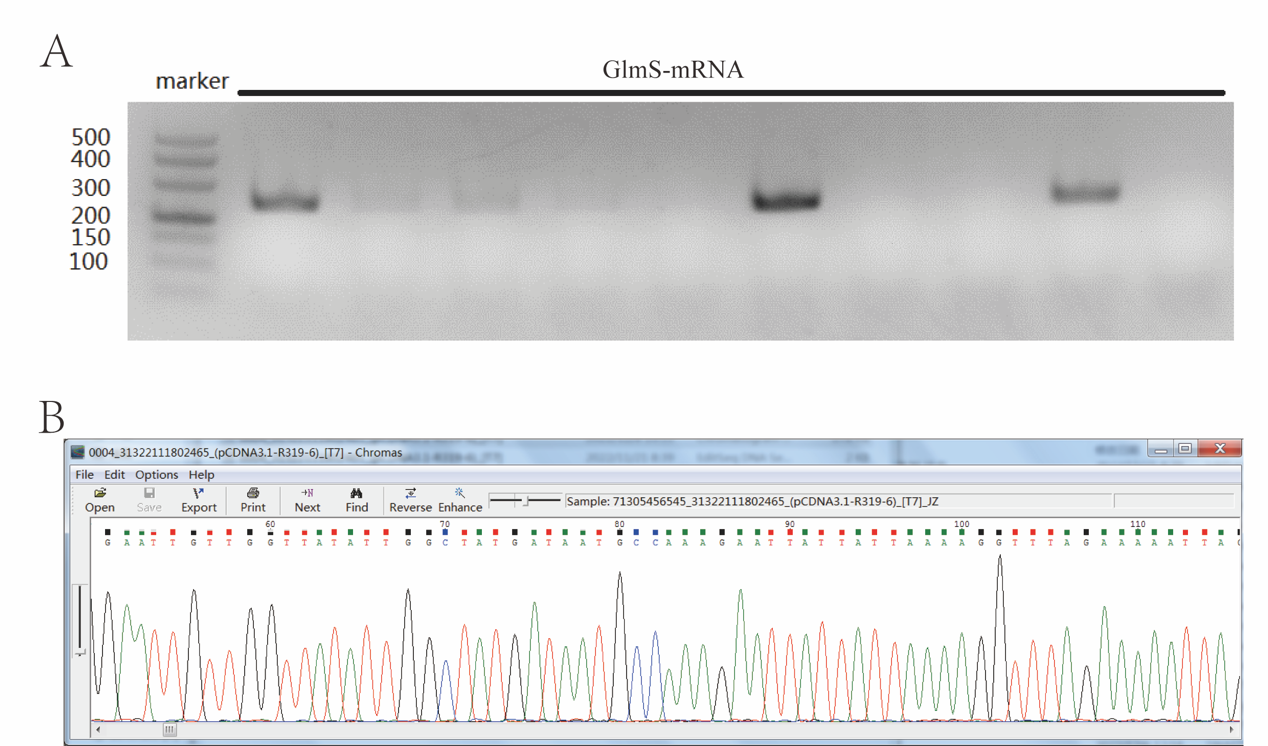


Supplementary Figure 5. Identifications of GlmS protein recombinant products. (A) Electropherogram of PCR products. (B) Sequencing results of PCR products.


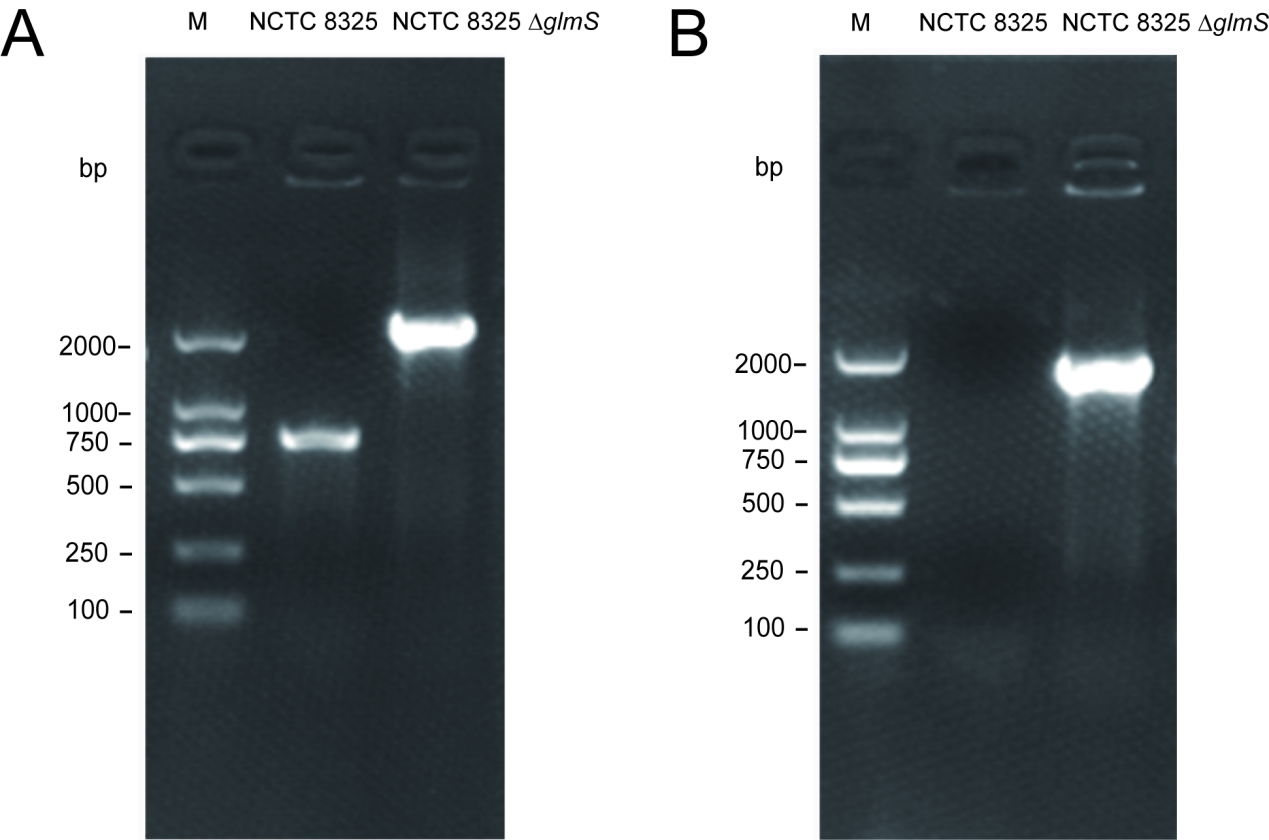


Supplementary Figure 6. Electrophoretic analysis of PCR amplification products of identification primers of wild and knockout strains. (A) *glmS*-JD-F/JD-R primer identification of wild and knockout strains. (B) *glmS-ter-F/R* primer identification of wild and knockout strains.


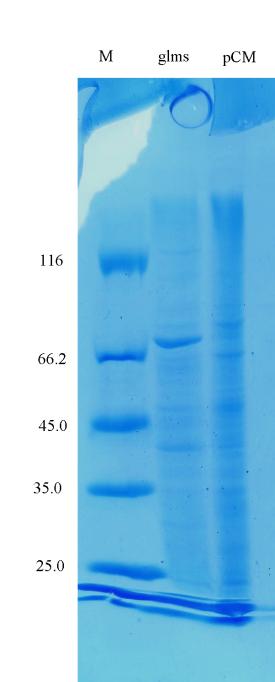


Supplementary Figure 7. SDS-PAGE of *glmS* overexpressing strain and pCM strain.

**2.2 Supplementary Tables**

Supplementary Table 1. PCR amplification system of Dual-luciferase assay

| Reagent | Volume (uL) |
| --- | --- |
| Template | 2 |
| Forwardprimer(10µM) | 2 |
| Reverseprimer(10µM) | 2 |
| Hieff Canace® Gold High-Fidelity DNA Polymerase(2 U/μL) | 1 |
| 2×Canace® Gold PCR buffer（including Mg2+，dNTPs） | 25 |
| ddH2O | 18 |

Supplementary Table 2. PCR procedures of Dual-luciferase assay

| Temperature | Time | Circles |
| --- | --- | --- |
| 98℃ | 5min | 1 |
| 98℃ | 10s | 35 |
| 65℃ | 30s |  |
| 72℃ | 1kb/min |  |
| 72℃ | 5min | 1 |

Supplementary Table 3. Enzymatic system

| Reagent |  | Volume (ul) |
| --- | --- | --- |
| PGL4.10 |  | 900-1500ng |
| Fastdigest XhoI |  | 1 |
| Fastdigest Hind III |  | 1 |
| 10×universeBuffer |  | 3 |
| ddH2O |  | To30 |

Enzymatic procedure was incubated at 37 ℃ for 3 hours.

Supplementary Table 4. Homologous recombination system

| Reagent | Volume (μL) |
| --- | --- |
| Linearized vector | X ul |
| Inserted fragment | Y ul |
| 2×Hieff Clone Enzyme Premix | 10 ul |
| ddH2O | To 20 |

X = [0.02 × the base pair numbers of vector] ng (0.03 pmol)

Y= [0.04 × the base pair numbers of inserted fragment] ng (0.06 pmol) or = [0.06 × the base pair numbers of inserted fragment] ng (0.09 pmol)

Note: When the length of the inserted fragment is greater than that of the vector, the calculation method for the vector and inserted fragment dosage should be interchanged.

Supplementary Table 5. Primers for each fragment of SigB promoter in the promoter truncation test.

| Gene name | Primer sequence (5'--3'） | bp |
| --- | --- | --- |
| Site 1-F | CCTGAGCTCGCTAGCCTCGAGTTGCAAACGACAAAATTGATAAGTGCAATTAAAT | 400 |
| Site 1-R | CAGTACCGGATTGCCAAGCTTAGCTAAGTCTATCTCTTTATCGTGAACTTG |  |
| Site 2-F | CCTGAGCTCGCTAGCCTCGAGTTCTAGCTTACAACAAACAATGCTTAAAAC | 400 |
| Site 2-R | CAGTACCGGATTGCCAAGCTTATTCTTCTTTTTCAGCGCGATAAATATATCC |  |
| Site 3-F | CCTGAGCTCGCTAGCCTCGAGTTTGAAGAAATTTCAGTTAGAGGTAGAGTGT | 400 |
| Site 3-R | CAGTACCGGATTGCCAAGCTTTATTAAAAATATTTATCTTCATTTTAAGCGCTGTATC |  |
| Site 4-F | CCTGAGCTCGCTAGCCTCGAGAAGTAGGAGTGTAATGAAATGAATCTTAATATAG | 400 |
| Site 4-R | CAGTACCGGATTGCCAAGCTTCATACTCTGCCGATGCTGGCACG |  |
| Site 5-F | CCTGAGCTCGCTAGCCTCGAGGTAAGTTTAATTCGTTTAACACTTTCTGGC | 400 |
| Site 5-R | CAGTACCGGATTGCCAAGCTTTATTTCGCACCTGCTCTTTTTTTATATACTTA |  |

Supplementary Table 6. The primer sequences used in qRT-PCR.

| Gene name | Primer sequence (5'--3'） |
| --- | --- |
| 16S rRNA-F | GAACCGCATGGTTCAAAAGT |
| 16S rRNA-R | TATGCATCGTTGCCTTGGTA |
| *glmS*-F | CAATGTTACAAGTGACAAGC |
| *glmS*-R | CATTACTGCTGGTTGTTCAT |
| *sigB*-F | AGTGTACATGTTCCGAGACG |
| *sigB*-R | GTCCCATTTCCATTGCTTC |
| *hla*-F | CTGATTACTATCCAAGAAATTCGATTG |
| *hla*-R | CTTTCCAGCCTACTTTTTTATCAGT |
| *hlb*-F | GCCAAAGCCGAATCTAAG |
| *hlb*-R | GCGATATACATCCCATGGC |
| *hld*-F | AAGAATTTTTATCTTAATTAAGGAAGGAGTG |
| *hld*-R | TTAGTGAATTTGTTCACTGTGTCGA |
| *hlg*-F | TGGCTCATTCAACTACTC |
| *hlg*-R | TTCTATCAACGGCTAAAC |

Supplementary Table 7. The primer sequences used in Mutant construction.

| Gene name | Primer sequence (5'--3'） |
| --- | --- |
| *glmS*-19-F | TGTAAAACGACGGCCAGTTACTCTAACATTGCCATAGAAAA |
| *glmS*-19-R | CTATGACCATGATTACGCCACCAACCAACAAGTGGACCCATA |
| *glmS-*down-F | TGATTCTAAAAAAGTGAAAACGACATTTTCCTCCATATTG |
| *glmS*-up-R | TATGGAGGAAAATGTCGTTTTCACTTTTTTAGAATCAATC |
| *glmS*-pKOR1-F | CCTCGGAACCGGTACCACTCTAACATTGCCATAGAAAATC |
| *glmS-*pKOR1-R | GCAGTGAGCGCAACGCAATACCAACCAACAAGTGGACCCA |
| pKOR1-*glmS*-F | CACTTGTTGGTTGGTATTGCGTTGCGCTCACTGCCCGCTT |
| pKOR1-*glmS*-R | ATGGCAATGTTAGAGTGGTACCGGTTCCGAGGCTCAACGT |
| *glmS*-JD-F | CCAGCACCATTCAACCCATATAA |
| *glmS*-JD-R | GTTAAAGCGCCTGTGCAAATAAA |
| *glmS*-ter-F | CAAGGTTACGTGGTTTATCAACA |
| *glmS*-ter-R | ATGTGTGGAATTGTTGGTTATAT |
